# Dietary supplement use among cancer survivors and the general population: a nation-wide cross-sectional study

**DOI:** 10.1186/s12885-017-3885-1

**Published:** 2017-12-28

**Authors:** Sihan Song, Jiyoung Youn, Yun Jung Lee, Minji Kang, Taisun Hyun, YoonJu Song, Jung Eun Lee

**Affiliations:** 1 Department of Food and Nutrition, Seoul National University, Gwanak-ro 1, Gwanak-gu, Seoul, 08826 Republic of Korea; 2 Department of Food and Nutrition, Chungbuk National University, Chungdae-ro 1, Seowon-Gu, Cheongju, Chungbuk 28644 Republic of Korea; 3 Major of Food and Nutrition, School of Human Ecology, The Catholic University of Korea, 43 Jibong-ro, Wonmi-gu, Bucheon-si, Gyeonggi-do 14662 Republic of Korea

**Keywords:** Cancer survivors, Dietary supplement use, Korea National Health and Nutrition Examination Survey

## Abstract

**Background:**

Use of dietary supplements among cancer survivors is common and controversial, but information on the amount of nutrients from supplements among cancer survivors is limited. We examined the amount of nutrients and their contribution to total nutrient intake from supplements and compared these data between cancer survivors and cancer-free individuals. We also identified factors associated with supplement use among cancer survivors.

**Methods:**

We identified 400 cancer survivors and 10,387 cancer-free individuals, aged ≥ 19 years, from the fifth Korea National Health and Nutrition Examination Survey (KNHANES) V-1, 2 (2010, 2011). We calculated the amount of nutrients consumed from foods and supplements, the percent contributions of supplement nutrients to total nutrient intakes and cancer survivors’ nutrient intakes relative to the Estimated Average Requirements (EARs) and the Tolerable Upper Intake Levels (ULs). We examined factors associated with supplement use among cancer survivors.

**Results:**

We found that 33.3% of cancer survivors and 22.1% of cancer-free individuals reported the use of dietary supplements. Compared to cancer-free individuals, cancer survivors had higher intakes of riboflavin, folate, and iron from foods (*p* < 0.05 for each), and higher intakes of calcium (*p* = 0.05) and vitamin C (*p* = 0.01) from foods and supplements. The similar pattern was observed for the percent contributions to total nutrient intake. Cancer survivors had higher proportion of participants below EARs than cancer-free individuals for thiamin and niacin (*p* < 0.05 for each). The proportions of cancer survivors below the EARs were 61.2% for calcium, 49.1% for riboflavin, and 43.5% for folate and the proportions of cancer survivors above the ULs were 3.3% for iron, and 2.3% for vitamin A. For female cancer survivors, education above an elementary school level, moderate physical activity, low vegetable intake, and high circulating vitamin D levels were associated with supplement use. For male cancer survivors, living in an urban area, no consumption of alcohol, and lower energy intake, were associated with supplement use.

**Conclusions:**

Korean cancer survivors have higher rate of dietary supplement use and higher contribution from supplements to total nutrient intake than cancer-free individuals. Demographic and lifestyle factors were associated with supplement use among cancer survivors.

**Electronic supplementary material:**

The online version of this article (10.1186/s12885-017-3885-1) contains supplementary material, which is available to authorized users.

## Background

Cancer survivors tend to start taking dietary supplements after receiving a diagnosis of cancer [1–4]. Previous studies have reported that, among cancer survivors, a high desire for personal control was associated with taking new dietary supplements [1] and the most commonly reported reasons for dietary supplement use were to improve health (e.g., immune system) and prevent disease [3, 4]. A previous systematic review reported a tendency for a higher prevalence of vitamin and mineral supplement use among cancer survivors (64–81%) than general US adults (52%) [5]. Furthermore, according to this review, the prevalence of dietary supplement use was relatively higher in breast cancer survivors than other cancer sites, and high education levels and female sex were associated with dietary supplement use among cancer survivors [5].

Dietary supplement use in cancer care remains controversial [6–8]. Cancer patients who lost significant weight before radiotherapy or chemotherapy treatment are often offered nutritional supplements along with enteral tube feeding [9], whereas there are concerns for herb-drug interactions and potential detrimental effects of antioxidants on cancer treatment [6–8]. The American Cancer Society guidelines suggested that cancer survivors should assess their nutrient deficiency first, and avoid to take excessive amount of nutrients from dietary supplements [7]. Given limited research on the amount of nutrients consumed from dietary supplements among cancer survivors, it is important to examine whether their nutrient levels are adequate.

However, only a few survey studies have reported the amount of nutrients or the contribution of dietary supplements to total nutrient intakes among cancer survivors. A French study calculated nutrient intakes from foods and supplements among 270 cancer survivors, and found that the contribution of vitamin or mineral supplement to total nutrient intakes ranged from 0% for sodium and potassium to 79% for vitamin D [3]. A US study of 753 cancer survivors reported that the proportion consuming amounts below Estimated Average Requirements (EARs) among 559 supplement users was substantially reduced after the addition of nutrient intakes from dietary supplements: vitamin E (81% to 12%), magnesium (77% to 40%), vitamin C (47% to 9%), and vitamin A (45% to 8%) [10]. In that study, the proportion consuming amounts above Tolerable Upper Intake Levels (ULs) was less than 10% [10]. Another US study of 435 breast cancer survivors also found that less than 5% of 352 dietary supplement users consumed nutrient intakes exceeding ULs [11].

Cancer is the leading cause of death in South Korea [12]. The estimated cumulative risk of developing cancer during a lifetime is 36.2%, and thyroid cancer is the most commonly diagnosed cancer, followed by stomach, colorectal, lung, and breast cancer, as reported in 2014 [13]. Dietary supplement use is also common among Korean adults. The age-standardized prevalence of dietary supplement use for at least 2 weeks during the past year among Korean adults increased from 25.7% in 2005 to 41.5% in 2015 [14]. Several studies found that dietary supplements were commonly used among Korean cancer patients: the proportion of dietary supplement use was 53.2% and 78.1% among 126 and 105 gastrointestinal cancer patients, respectively [15, 16], and the proportion of vitamin/mineral use was 24.1% and 27.1% among 339 and 288 breast cancer patients, respectively [17, 18]. However, to our knowledge, the amount of nutrients consumed and the nutritional contribution of dietary supplement to total nutrient intakes have not been reported among Korean cancer survivors. No information on the amount or contribution of nutrients consumed from dietary supplements among Korean cancer survivors may preclude researchers and health professionals from considering further investigation or intervention on cancer survivor’s supplement use.

Therefore, we aimed to examine dietary supplement intake and its contribution to nutrient intake among cancer survivors using a database of dietary supplements, which is part of the fifth Korea National Health and Nutrition Examination Survey (KNHANES V-1,2), 2010 and 2011, nationwide database [19, 20]. We calculated the nutritional contribution of dietary supplements among the general population to compare with cancer survivors. Furthermore, we identified the characteristics of dietary supplement users among Korean cancer survivors.

## Methods

### Data source and study population

We included participants of the KNHANES V-1,2 (2010, 2011), a population-based cross-sectional nation-wide survey [20]. The KNHANES is conducted annually by the Korea Centers for Disease Control and Prevention (KCDC) to assess the health and nutritional status of Koreans. Sampling of non-institutionalized Korean citizens occurs via a multistage clustered probability design, and the health and nutritional information of participants is gained from health interviews, clinical examinations, and nutrition surveys. A detailed description of the study is provided elsewhere [21]. The health interviews and examinations were conducted in the mobile examination center (MEC). Information on smoking, alcohol use, and physical activity was collected via a self-administered questionnaire. Housing characteristics and details of medical conditions and socioeconomic status were obtained by face-to-face interviews. Body mass index (BMI, kg/m^2^) was calculated using weight and height, which were measured during the health examination. Fasting blood serum and urine samples were collected from participants aged ≥ 10 years to obtain biochemical profiles. Dietary supplement use and dietary intake were obtained by face-to-face interviews conducted in the homes of the participants [21]. We used data from one-day 24-h dietary recalls to estimate nutrient intake from foods and dietary supplements. Energy and nutrient intakes from foods were calculated using the Korean National Rural Living Science Institute (KNRLSI) and Korea Health Industry Development Institute (KHIDI) databases [20, 22]. We estimated folate intake from 24-h dietary recalls using the folate database developed by Kim JH et al. [23] and Yon M et al. [24]. Non-quantitative food frequency questionnaires (FFQs) were used to estimate the daily servings of vegetables, fruits, and red and processed meats.

Of 17,476 participants identified from the KNHANES V-1, 2 (2010–2011) survey, we excluded those with 1) age < 19 years, 2) no information on 24-h dietary recalls, 3) no information from a physician regarding cancer diagnosis, or 4) implausible energy intake (above the log-transformed mean ± 3SD). As a result, a total of 10,787 adults aged ≥ 19 years were eligible for the study. All participants signed an informed consent form. Ethics approval was obtained from the KCDC Institutional Review Board (IRB) Ethics Committee (2010-02CON-21-C, 2011-02CON-06-C) [20].

### Ascertainment of cancer diagnosis

Trained interviewers asked the participants, during face-to-face health interviews, whether they had ever been diagnosed with cancer by a physician. The participants reporting a history of cancer were asked for the site of cancer and the age when cancer was diagnosed [21]. We calculated survival time since the diagnosis of cancer by subtracting the age at the first diagnosis of cancer from the current age.

### Calculation of the nutrient amounts from dietary supplements

We defined dietary supplement use as the use of any dietary supplement on the day preceding the date of the survey. The participants were otherwise categorized as non-users. Trained interviewers asked the participants about their dietary supplement use, including type, product name, manufacturing company, distributor, frequency, and amount, when they administered the 24-h dietary recalls during the face-to-face interview [19]. Using the dietary supplement database, we calculated the nutrient amounts from supplements [19]. This database was developed by identifying the composition of nutritional supplements through searching a medication database, the Korean Ministry of Food and Drug Safety (KMFDS) database, or product distributors [19]. The KCDC has publicly released the nutrient values of calcium, phosphorus, iron, vitamin A, thiamine, riboflavin, niacin, and vitamin C from the intake of dietary supplements in the KNHANES V-1, 2 (2010–2011). In this study, we developed a database of folate from dietary supplements developed using the same procedure [19].

### Statistical analysis

We applied sampling weight to account for the complex, multistage sampling design of the KNHANES using the SAS survey procedures. Sampling weights were divided by the number of combined data to obtain estimates representative of the Korean population on average from 2010 to 2011 [20]. Descriptive statistics were estimated using PROC SURVEYFREQ for categorical variables and PROC SURVEYMEANS for continuous variables. We identified the proportion (%) of dietary supplement use of the study population according to the cancer sites of cancer survivors or types of dietary supplements. We presented 10 cancer sites that had enough cancer survivors and combined other cancer sites. We compared the proportion of dietary supplement use between cancer survivors and cancer-free individuals using the Rao-Scott chi-square test. We used PROC SURVEYREG to examine whether nutrient intakes from foods only and total (foods + supplements) differed between cancer survivors and cancer-free individuals. Nutrient intakes were natural logarithm transformed to obtain a normal distribution. We obtained the Least-squares means (LS-means) and 95% confidence intervals (95% CIs) adjusting for age (continuous, years), sex (men, women), energy intake (continuous, kcal/day), BMI (continuous, kg/m^2^), and education level (elementary school or below, middle school, high school, college or above). The percent contribution for each participant was calculated by dividing the nutrient intakes from dietary supplements by the total nutrient intakes from foods and dietary supplements. We calculated the mean and standard error (SE) of nutrient intake and of the percent contribution of nutrient intake from dietary supplements to the total intake for cancer survivors and cancer-free individuals. We also identified the proportions of nutrient intakes below the EARs and above the ULs for nutrient intakes of foods and total (foods + supplements) intakes among both cancer survivors and cancer-free individuals. We used the EARs and ULs established by Dietary Reference Intakes for Koreans (KDRIs) 2010 [25]. We compared the proportions below the EARs between cancer survivors and cancer-free individuals using the Rao-Scott chi-square test.

We used PROC SURVEYLOGISTIC to obtain the Odds Ratios (ORs) and 95% CIs and compare the demographic, lifestyle, and clinical characteristics of supplement users and non-users among cancer survivors. Because we found a sex difference in the characteristics of supplement users, we conducted separate analyses for men and women. In the logistic regression models, we adjusted for age (continuous, years), energy intake (continuous, kcal/day), residential area (rural, urban), and education level (elementary school or below, middle school, high school, college or above). To test for trends, we included the ordinal variable or median values of exposure to the models. To consider the answers on the general questionnaire of dietary supplement use (any supplement use of > 1 per week over the preceding month; yes/no), we conducted a sensitivity analysis. We found that the results were similar when we excluded those with discrepancies in the responses between the 24-h dietary recalls and the general questionnaire (data not shown). To test for a potential confounding effect of time since cancer diagnosis, we conducted a sensitivity analysis by additionally adjusting for cancer sites with a > 90% 5-year survival rate in South Korea (thyroid, breast and prostate cancers), and it resulted in virtually unchanged estimates (data not shown). The level of statistical significance was a *p*-value < 0.05 in two-sided tests. We used SAS version 9.3 (SAS Institute Inc., Cary, NC, USA) for all statistical analyses.

## Results

We found that 33.3% of cancer survivors and 22.1% of cancer-free individuals reported using dietary supplements (*p* < 0.001) (Table 1). Cancer survivors also had a higher rate of dietary supplement use than cancer-free individuals among female (*p* = 0.001) and male (*p* = 0.04). For female cancer survivors, the dietary supplement use rate was the highest in breast cancer survivors (55.9%), followed by lung (49.6%), stomach (38.3%), cervix or corpus uteri (38.1%), and thyroid cancer survivors (31.7%). For male cancer survivors, the dietary supplement use rate was the highest in thyroid cancer survivors (31.6%), followed by bladder (31.5%), liver (28.5%), colorectal (26.6%), and stomach (23.9%) cancer survivors. The frequency of dietary supplement use without sampling weight is presented in Additional file 1: Table S1.

**Table 1:** Dietary supplement use of the study population and according to cancer sites of cancer survivors^a^

	All		Female		Male	
	Number	Dietary supplement use % (SE)	Number	Dietary supplement use % (SE)	Number	Dietary supplement use % (SE)
Cancer-free individuals	10,387	22.1 (0.6)	6154	26.0 (0.7)	4233	18.1 (0.8)
Cancer survivors	400	33.3 (2.8)	260	37.0 (3.6)	140	26.7 (4.7)
*P* value^b^	< 0.001		0.001		0.04	
By cancer site						
Stomach	71	29.8 (6.1)	27	38.3 (11.4)	44	23.9 (7.0)
Cervix or corpus uteri	67	38.1 (7.2)	67	38.1 (7.2)	0	–
Thyroid	63	31.7 (6.5)	54	31.7 (7.1)	9	31.6 (16.3)
Breast	59	55.9 (7.0)	59	55.9 (7.0)	0	–
Colorectal	52	22.5 (5.9)	27	18.5 (8.1)	25	26.6 (8.6)
Bladder	14	28.7 (4.0)	5	23.1 (0.0)	9	31.5 (5.9)
Lung	13	23.0 (11.4)	4	49.6 (28.3)	9	15.7 (5.4)
Prostate	12	19.4 (7.0)	0	–	12	19.4 (7.0)
Liver	11	25.8 (6.4)	2	0.0	9	28.5 (7.1)
Renal	9	0.0	4	0.0	5	0.0
Others^c^	46	29.2 (6.0)	20	25.1 (12.7)	26	32.0 (8.4)

^a^Cancer survivors with multiple cancer sites were counted multiple times; 11 participants had been diagnosed with cancer at two sites, and 3 participants had been diagnosed with cancer at three sites among the 400 cancer survivors

^b^Rao-Scott chi-square *p* values were obtained using PROC SURVEYFREQ to compare the proportion of dietary supplement use between cancer survivors and cancer-free individuals

^c^Others included 19 cancer sites (e.g., larynx, lymphoma, skin, ovarian, and esophageal)

The major types of dietary supplements consumed by cancer survivors and cancer-free individuals were similar (Table 2). Multi-vitamins and minerals were most commonly used by cancer survivors (24.6% among dietary supplement users), followed by vitamin C (18.7%), omega-3/fish oil (14.8%), red ginseng (10.7%), and calcium (4.4%). Cancer-free individuals also commonly used multi-vitamins and minerals (33.5% among dietary supplement users), omega-3/fish oil (17.5%), vitamin C (13.3%), red ginseng (9.0%), and glucosamine/chondroitin (4.0%).

**Table 2:** Top 10 dietary supplements commonly used among dietary supplement users^a^

Cancer survivors (*n* = 141)		Cancer-free individuals (*n* = 2651)	
Supplement type	% (SE)	Supplement type	% (SE)
Multi-vitamin/minerals	24.6 (4.5)	Multi-vitamin/minerals	33.5 (1.3)
Vitamin C^b^	18.7 (4.8)	Omega-3/fish oil	17.5 (1.0)
Omega-3/fish oil	14.8 (3.8)	Vitamin C^b^	13.3 (0.9)
Red ginseng	10.7 (4.3)	Red ginseng	9.0 (0.8)
Calcium^c^	4.4 (2.2)	Glucosamine/Chondroitin	4.0 (0.5)
Yeast	4.2 (3.0)	Calcium^c^	3.7 (0.4)
Glucosamine/Chondroitin	2.4 (1.0)	Evening primrose oil	2.3 (0.4)
Garlic extract	1.9 (1.3)	Vitamin Bs^d^	1.1 (0.2)
Mycelial culture extract from phellinus linteus	1.5 (1.5)	Vitamin E	1.1 (0.2)
Octacosanol	1.0 (1.0)	Vitamin A/Beta-carotene	0.9 (0.2)

^a^Cancer survivors who used more than one dietary supplement were counted multiple times

^b^Vitamin C and Vitamin C (major component) + Vitamin E

^c^Calcium, Calcium (major component) + Vitamin D, and Calcium (major component) + Vitamin D + Magnesium

^d^Thiamine, Vitamin B6, Folate, Vitamin B12, Folate + Vitamin B12, and Vitamin B complex

We compared nutrient intakes from foods and total (foods + supplements) between cancer and cancer-free individuals (Table 3). For nutrient intakes from foods, cancer survivors had higher amounts of iron (*p* = 0.04), riboflavin (*p* = 0.01), and folate (*p* = 0.01) compared to cancer-free individuals. For total nutrient intakes (foods + supplements), cancer survivors had higher amount of iron (*p* = 0.04), riboflavin (*p* = 0.03), folate (*p* = 0.02), calcium (*p* = 0.05), and vitamin C (*p* = 0.01) compared to cancer-free individuals. Among dietary supplement users, there were no significant differences between cancer survivors and cancer-free individuals for both nutrient intakes from foods and total (foods + supplements).

**Table 3:** Nutrient intakes from food and total (foods and supplements) among cancer survivors and cancer-free individuals

LS means (95% CI)^a^	Food only		*P* value^b^	Foods + Supplements		*P* value^b^
	Cancer survivors	Cancer-free individuals		Cancer survivors	Cancer-free individuals	
All (n)	400	10,387		400	10,387	
Calcium (mg/d)	437.6 (400.9–477.6)	413.6 (407–420.2)	0.21	465.0 (425.1–508.7)	424.8 (418.0–431.7)	0.05
Phosphorus (mg/d)	1083.0 (1040.9–1126.9)	1056.9 (1049.7–1064.0)	0.23	1088.0 (1045.4–1132.2)	1060.0 (1052.9–1067.2)	0.21
Iron (mg/d)	13.3 (12.4–14.3)	12.3 (12.1–12.5)	0.04	13.9 (12.8–15.0)	12.8 (12.6–13.0)	0.04
Vitamin A (μg RE/d)	552.3 (491.8–620.2)	531.0 (516.3–546.2)	0.51	582.9 (517.7–656.4)	560.9 (544.7–577.6)	0.53
Thiamine (mg/d)	1.2 (1.1–1.2)	1.1 (1.1–1.1)	0.09	1.4 (1.3–1.5)	1.3 (1.3–1.3)	0.35
Riboflavin (mg/d)	1.1 (1.0–1.1)	1.0 (1.0–1.0)	0.01	1.2 (1.1–1.3)	1.1 (1.1–1.2)	0.03
Niacin (mg/d)	14.9 (14.3–15.5)	14.4 (14.3–14.5)	0.10	16.4 (15.4–17.4)	15.6 (15.4–15.8)	0.11
Folate (μg DFE/d)	335.7 (316.7–355.8)	308.6 (304.4–312.8)	0.01	358.5 (334.8–383.9)	330.9 (325.8–336.2)	0.02
Vitamin C (mg/d)	87.9 (77.4–99.8)	77.8 (76.1–79.6)	0.06	111.5 (96.4–128.9)	91.4 (89.0–93.9)	0.01
Among users (n)	141	2651		141	2651	
Calcium (mg/d)	443.5 (396.3–496.5)	440.2 (425.9–454.9)	0.89	532.5 (470.4–602.7)	492.7 (475.7–510.4)	0.22
Phosphorus (mg/d)	1080.4 (1022.9–1141.2)	1092.9 (1078.7–1107.4)	0.68	1096.3 (1037.9–1158.1)	1107.7 (1093.0–1122.4)	0.71
Iron (mg/d)	13.7 (12.5–15.1)	13.1 (12.6–13.5)	0.34	15.5 (13.7–17.4)	15.2 (14.6–15.8)	0.81
Vitamin A (μg RE/d)	543.9 (468.6–631.2)	567.0 (539.2–596.2)	0.59	645.3 (554.4–751.1)	718.8 (678.5–761.4)	0.17
Thiamine (mg/d)	1.2 (1.1–1.3)	1.1 (1.1–1.2)	0.13	2.2 (1.7–2.8)	2.4 (2.2–2.5)	0.56
Riboflavin (mg/d)	1.0 (1.0–1.1)	1.0 (1.0–1.1)	0.92	1.8 (1.5–2.2)	1.9 (1.8–2.0)	0.63
Niacin (mg/d)	14.7 (13.8–15.6)	14.7 (14.4–14.9)	0.99	20.1 (17.4–23.1)	21.0 (20.2–21.9)	0.54
Folate (μg DFE/d)	333.3 (306.1–362.9)	321.3 (313.8–329)	0.41	409 (359.3–465.7)	434.8 (420.4–449.8)	0.37
Vitamin C (mg/d)	86.0 (73.7–100.4)	82.8 (79.6–86.2)	0.64	185.7 (145.2–237.3)	170.6 (160.4–181.4)	0.51

Abbreviations: *LS means* Least squares means, *95% CI* 95% confidence interval, *RE* retinol equivalent, *DFE* dietary folate equivalent

^a^Least square means adjusted for age (continuous, years), sex (men, women), energy intake (continuous, kcal/day), body mass index (continuous, kg/m^2^), and education level (elementary school or below, middle school, high school, and college or above) were obtained using PROC SURVEYREG

^b^ *P* values were obtained based on the Wald’s F test

**Fig. 1:**
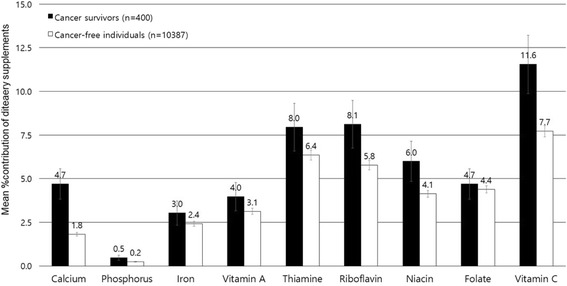
The percent contribution from dietary supplements (Mean ± SE) to total nutrient intakes among overall participants (400 cancer survivors and 10,387 cancer-free individuals)

**Fig. 2:**
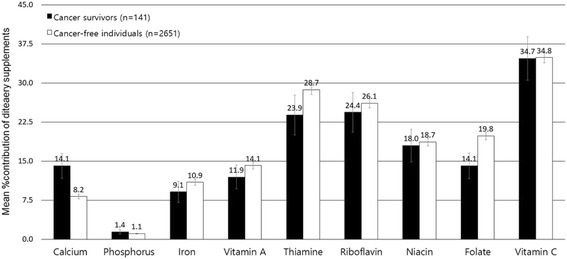
The percent contribution from dietary supplements (Mean ± SE) to total nutrient intakes among dietary supplement users (141 cancer survivors and 2651 cancer-free individuals)

Compared to cancer-free individuals, cancer survivors tended to have higher contributions of dietary supplements to total nutrient intakes among all participants (Fig. 1). The contribution of dietary supplements to total nutrient intakes ranged from 0.5% for phosphorus to 11.6% for vitamin C among cancer survivors, whereas it ranged from 0.2% for phosphorus to 7.7% for vitamin C among cancer-free individuals. The percent contribution of dietary supplements to total calcium intakes among cancer survivors (4.7%) was 2.6 times higher than that among cancer-free individuals (1.8%). Calcium and vitamin C showed a relatively higher difference of the percent contribution of dietary supplements to total intakes between cancer survivors and cancer-free individuals than other nutrients. Among supplement users, the contribution of dietary supplement to total nutrient intakes were similar between cancer survivors and cancer-free individuals, except for calcium (Fig. 2).

We found that a higher proportion of cancer survivors consumed thiamin (*p* = 0.02) and niacin (*p* = 0.03) from foods and supplements below the EARs compared to cancer-free individuals (Table 4). Among dietary supplement users, cancer survivors had a higher proportion of individuals whose intakes of vitamin A (*p* = 0.03), thiamin (*p* = 0.01), riboflavin (*p* = 0.01), and folate (*p* = 0.01) from foods and supplements were below the EARs compared to cancer-free individuals. Overall, both cancer survivors and cancer-free individuals showed that more than 60% consumed calcium from foods and supplements below the EARs. The proportions of study population who consumed nutrients below the EARs from foods were reduced after the addition of nutrient intakes from dietary supplements. Among all participants, the proportions of cancer survivors who consumed nutrients below the EARs ranged from 31.6% (niacin) to 49.1% (riboflavin) for six nutrients (vitamin A, thiamin, riboflavin, niacin, folate, and vitamin C) from foods and supplements; for cancer-free individuals, more than 30%, but less than 50%, consumed levels of vitamin A, riboflavin, folate, and vitamin C below the EARs from foods and supplements. Among dietary supplement users, the proportions of consumption below EARs were reduced after adding nutrient intake from supplement to that from foods only by an average of 24.3% among cancer survivors and 30.3% among cancer-free individuals with the highest reduction for vitamin C.

**Table 4:** The proportions of below estimated average requirement (EAR) among cancer survivors and cancer-free individuals

Nutrient	Cancer survivors			Cancer-free individuals			*P* value^b^	*P* value^c^
	% Below EAR (SE)			% Below EAR (SE)				
	Food only	Foods and supplements	% Change^a^	Food only	Foods and supplements	% Change^a^		
All (n)	400			10,387				
Calcium (mg/d)	64.2 (3.2)	61.2 (3.1)	4.7	64.7 (0.7)	63.0 (0.7)	2.6	0.88	0.57
Phosphate (mg/d)	9.8 (1.8)	9.7 (1.8)	1.0	7.9 (0.3)	7.9 (0.3)	0.0	0.27	0.28
Iron (mg/d)	18.6 (2.5)	17.2 (2.5)	7.5	22.1 (0.5)	21.1 (0.5)	4.5	0.20	0.15
Vitamin A (μg RE/d)	44.0 (3.1)	40.3 (3.1)	8.4	37.3 (0.7)	35.6 (0.7)	4.6	0.03	0.11
Thiamin (mg/d)	40.4 (3.2)	36.3 (3.2)	10.2	32.1 (0.6)	29.3 (0.6)	8.7	0.01	0.02
Riboflavin (mg/d)	56.3 (3.1)	49.1 (3.1)	12.8	49.4 (0.7)	45.6 (0.7)	7.7	0.02	0.25
Niacin (mg/d)	35.4 (3.0)	31.6 (2.7)	10.7	27.8 (0.6)	25.7 (0.6)	7.6	0.01	0.03
Folate (μg/d)	46.8 (3.3)	43.5 (3.4)	7.1	47.0 (0.7)	43.7 (0.6)	7.0	0.93	0.94
Vitamin C (mg/d)	42.2 (3.2)	35.9 (3.0)	14.9	42.3 (0.7)	37.8 (0.7)	10.6	0.96	0.51
Among users (n)	141			2651				
Calcium (mg/d)	68.7 (5.0)	59.5 (5.2)	13.4	58.7 (1.3)	50.8 (1.3)	13.5	0.06	0.11
Phosphate (mg/d)	7.0 (2.7)	6.7 (2.7)	4.3	5.7 (0.6)	5.5 (0.6)	3.5	0.63	0.64
Iron (mg/d)	19.5 (4.6)	15.1 (4.2)	22.6	18.2 (1.0)	13.6 (0.9)	25.3	0.79	0.71
Vitamin A (μg RE/d)	46.4 (5.4)	35.4 (5.1)	23.7	33.0 (1.2)	24.9 (1.1)	24.6	0.01	0.03
Thiamin (mg/d)	42.9 (5.5)	30.7 (5.5)	28.4	30.8 (1.2)	18.1 (1.0)	41.2	0.02	0.01
Riboflavin (mg/d)	63.7 (5.3)	42.1 (5.3)	33.9	45.3 (1.3)	28.2 (1.0)	37.8	0.001	0.01
Niacin (mg/d)	36.4 (5.8)	25.0 (5.0)	31.3	26.4 (1.1)	16.6 (0.9)	37.1	0.06	0.05
Folate (μg/d)	52.7 (5.5)	42.8 (5.6)	18.8	43.3 (1.3)	28.4 (1.1)	34.4	0.10	0.01
Vitamin C (mg/d)	44.6 (5.4)	25.7 (4.5)	42.4	38.6 (1.3)	18.4 (0.9)	52.3	0.27	0.07

Abbreviations: *RE* retinol equivalent, *DFE* dietary folate equivalents

^a^Calculated by dividing subtracted values (% below EAR from foods only – foods and supplements) by % below EAR from foods only

^b^Rao-Scott chi-square *p* values were obtained using PROC SURVEYFREQ to compare the proportion of below EAR from foods between survivors and cancer-free individuals

^c^Rao-Scott chi-square *p* values were obtained using PROC SURVEYFREQ to compare the proportion of below EAR from foods and supplements between survivors and cancer-free individuals

A small proportion of cancer survivors consumed nutrient intakes exceeding ULs, even after the addition of intakes from supplements (Additional file 2: Table S2). The proportions of consumption above ULs for nutrient intakes from foods and supplements ranged from 0.3% for phosphorus to 3.3% for iron among cancer survivors and ranged 0.2% for calcium to 2.9% for vitamin A among cancer-free individuals. Among dietary supplement users, the proportions of consumption above ULs were less than 4% among cancer survivors and less than 6% among cancer-free individuals.

We examined whether demographic, lifestyle, and clinical factors were associated with dietary supplement use among cancer survivors (Table 5). Among female cancer survivors, dietary supplement use was associated with education level: the ORs (95% CIs) were 4.75 (95% CI = 1.66–13.56) for middle school vs. elementary school or below and 4.51 (95% CI = 1.69–12.06) for high school vs. elementary school or below. Female cancer survivor supplement users were more likely to engage in moderate physical activity (OR = 3.95; 95% CI = 1.16–13.44 for yes vs. no) and were less likely to consume vegetables (OR = 0.81; 95% CI = 0.70–0.94 for one increment in one serving size of vegetable intake) compared to non-users. Among male cancer survivors, supplement users were less likely to live in rural areas (OR = 0.26; 95% CI = 0.08–0.84 for rural vs. urban) and were more likely to have lower total energy consumption (OR = 0.93; 95% CI = 0.88–0.99 for 100 kcal/d increment in energy intake) compared to non-users. Moreover, among male cancer survivors, supplement users were more likely to be never alcohol drinkers (OR = 7.88; 95% CI = 1.45–42.82 for never alcohol drinkers vs. ever alcohol drinkers) compared to non-users. We also examined the associations between dietary supplement use and blood levels of fasting blood glucose, total and high-density lipoprotein (HDL) cholesterol, triglycerides, and 25-hydroxyvitamin D3 (25(OH) D3). The use of dietary supplements was not significantly associated with these blood markers except for 25(OH) D3 among female cancer survivors; dietary supplement users tended to have higher 25(OH) D3 levels compared to non-users (OR = 1.27; 95% CI = 1.01–1.60 for a 5 ng/mL increment in 25(OH) D3).

**Table 5:** Odds ratios (ORs) and 95% confidence intervals (CIs)^a^ for supplement users vs non-users (*n* = 400)

Characteristics	Female cancer survivors				Male cancer survivors			
	Number	Non-use (*n* = 160)	Supplement use (*n* = 100)	Any supplement use vs. non-use	Number	Non-use (*n* = 99)	Supplement use (*n* = 41)	Any supplement use vs. non-use
		% (SE)	% (SE)	OR (95% CI)		% (SE)	% (SE)	OR (95% CI)
Age (years)								
19–59	134	56.7 (4.8)	68.6 (5.3)	Reference	37	49.3 (6.1)	41.4 (10.8)	Reference
60–69	74	22.9 (3.9)	19.5 (4.3)	0.95 (0.43–2.11)	49	24.8 (5.1)	28.3 (7.6)	1.97 (0.53–7.30)
≥ 70	52	20.4 (3.8)	11.9 (3.4)	0.85 (0.28–2.57)	54	25.8 (4.2)	30.4 (8.4)	1.35 (0.36–5.05)
*P* for trend				0.78				0.58
Marital status								
Married	252	93.5 (2.9)	97.8 (2.1)	Reference	134	88.0 (5.4)	89.8 (7.5)	Reference
Not married	8	6.5 (2.9)	2.2 (2.1)	0.24 (0.02–2.82)	6	12.0 (5.4)	10.2 (7.5)	0.61 (0.05–7.11)
Residential area								
Urban	202	77.0 (4.0)	79.6 (6.1)	Reference	99	65.8 (5.9)	86.3 (5.7)	Reference
Rural	58	23.0 (4.0)	20.4 (6.1)	1.04 (0.49–2.17)	41	34.2 (5.9)	13.7 (5.7)	0.26 (0.08–0.84)
Education level								
Elementary school or below	101	42.4 (4.6)	20.2 (4.3)	Reference	45	27.8 (4.5)	18.1 (6.3)	Reference
Middle school	48	15.4 (3.2)	25.5 (6.0)	4.75 (1.66–13.56)	16	5.9 (2.3)	15.6 (6.5)	5.08 (0.91–28.55)
High school	70	24.0 (4.5)	36.1 (6.0)	4.51 (1.69–12.06)	41	38.2 (6.7)	18.4 (6.4)	0.73 (0.18–2.87)
College or above	41	18.2 (4.1)	18.2 (4.9)	3.18 (0.92–11.04)	38	28.0 (6.0)	47.8 (10.3)	3.50 (0.78–15.75)
*P* for trend				0.07				0.15
Occupation^b^								
Unemployed	173	63.9 (4.8)	57.2 (6.9)	Reference	78	53.0 (6.3)	37.5 (8.8)	Reference
Employed	87	36.1 (4.8)	42.8 (6.9)	1.30 (0.65–2.60)	61	47.0 (6.3)	62.5 (8.8)	2.57 (0.98–6.75)
Equalized monthly household income^b^								
Low	67	26.3 (4.3)	18.6 (4.5)	Reference	46	27.4 (4.9)	29.6 (8.3)	Reference
Mid-low	62	25.5 (4.7)	25.3 (5.7)	1.14 (0.45–2.88)	35	24.7 (5.9)	26.0 (11.0)	1.49 (0.43–5.12)
Mid-high	59	26.2 (4.4)	18.9 (5.1)	0.87 (0.34–2.25)	31	24.6 (5.6)	23.2 (9.0)	0.67 (0.19–2.33)
High	68	22.1 (4.5)	37.2 (6.1)	1.71 (0.66–4.47)	26	23.3 (5.4)	21.2 (7.3)	1.02 (0.23–4.47)
*P* for trend				0.33				0.76
Body weight (kg)^b,c^	260	56.4 ± 0.9	59.1 ± 1.3	1.02 (0.99–1.06)	139	65.6 ± 1.8	65 ± 2.0	1.00 (0.95–1.05)
Body mass index^b^								
< 18.5	11	6.0 (2.6)	5.3 (3.5)	1.54 (0.33–7.11)	10	7.9 (4.1)	6.4 (3.4)	1.45 (0.21–10.18)
18.5–22.9	96	44.2 (5.2)	32.7 (5.9)	Reference	68	45.3 (6.0)	52.8 (10.6)	Reference
23–24.9	59	20.2 (3.5)	20.0 (4.7)	1.51 (0.68–3.36)	29	25.2 (5.1)	14.4 (5.8)	0.43 (0.10–1.93)
≥ 25	94	29.6 (4.4)	41.9 (6.3)	2.04 (0.93–4.50)	32	21.6 (5.5)	26.4 (9.0)	1.62 (0.41–6.42)
*P* for trend				0.17				0.93
Waist circumference (cm)^b,c^	259	78.3 ± 0.9	80.6 ± 1.4	1.03 (0.99–1.07)	139	82.9 ± 1.3	81.4 ± 1.6	0.98 (0.93–1.04)
Vigorous physical activity^b,d^								
No	231	88.1 (3.5)	87.6 (4.6)	Reference	112	78.8 (5.0)	73.7 (11.7)	Reference
Yes	27	11.9 (3.5)	12.4 (4.6)	1.00 (0.30–3.36)	27	21.2 (5.0)	26.3 (11.7)	0.95 (0.26–3.56)
Moderate physical activity^b,e^								
No	243	96.8 (1.2)	89.6 (5.1)	Reference	121	84.1 (4.7)	82.2 (10.9)	Reference
Yes	16	3.2 (1.2)	10.4 (5.1)	3.95 (1.16–13.44)	19	15.9 (4.7)	17.8 (10.9)	1.51 (0.32–7.03)
Smoking status^a^								
Never smoker	240	94.0 (2.4)	90.0 (3.5)	Reference	24	17.3 (5.4)	35.0 (11.1)	Reference
Ever smoker	19	6.0 (2.4)	10.0 (3.5)	2.15 (0.79–5.81)	116	82.7 (5.4)	65.0 (11.1)	0.35 (0.10–1.17)
Alcohol intake^a^								
Ever drinker	173	73.1 (4.2)	66.3 (6.2)	Reference	129	97.3 (1.6)	81.2 (10.8)	Reference
Never drinker	86	26.9 (4.2)	33.7 (6.2)	1.54 (0.66–3.59)	11	2.7 (1.6)	18.8 (10.8)	7.88 (1.45–42.82)
Energy intakes (100 kcal/day)^c^	260	16.7 ± 0.7	16.0 ± 0.6	0.96 (0.91–1.02)	140	22.2 ± 1.4	19.0 ± 1.1	0.93 (0.88–0.99)
Food intakes (serving/day)^b,c^								
Vegetables	253	4.5 ± 0.3	3.9 ± 0.2	0.81 (0.70–0.94)	131	4.2 ± 0.2	4.6 ± 0.5	1.12 (0.87–1.46)
Fruits	253	1.3 ± 0.1	1.4 ± 0.08	0.90 (0.64–1.27)	131	0.9 ± 0.06	1.1 ± 0.3	1.77 (0.82–3.81)
Red and processed meat	253	0.2 ± 0.02	0.3 ± 0.04	2.08 (0.45–9.56)	131	0.3 ± 0.03	0.3 ± 0.06	3.58 (0.18–70.93)
Time since cancer diagnosis^b,f^								
Less than 5 years	118	50.7 (5.2)	47.5 (6.0)	Reference	74	50.8 (6.1)	41.6 (9.5)	Reference
5 years or more	141	49.3 (5.2)	52.5 (6.0)	1.44 (0.71–2.89)	66	49.2 (6.1)	58.4 (9.5)	1.35 (0.53–3.43)
Chronic morbidity^g^								
No	122	52.4 (5.0)	62.9 (5.5)	Reference	70	62.4 (5.9)	55.8 (10.0)	Reference
Yes	138	47.6 (5.0)	37.1 (5.5)	0.93 (0.40–2.16)	70	37.6 (5.9)	44.2 (10.0)	1.19 (0.38–3.70)

^a^Models were adjusted for age (years, continuous), energy intakes (kcal/day, continuous), residential area (rural, urban), and education level (elementary school or below, middle school, high school, and college or above)

^b^Total number of participants was not equal to 260 for women or 140 for men because of some did not provide the relevant information

^c^Continuous variables are expressed as Mean ± SE

^d^Vigorous physical activity was defined as at least 20 min of vigorous-intensity activity on ≥ 3 days per week

^e^Moderate physical activity was defined as at least 30 min of moderate-intensity activity on ≥ 5 days per week

^f^Time since cancer diagnosis was calculated by subtracting the age at the first diagnosis of cancer from the current age

^g^Participants were categorized into yes if they answered to ever have hypertension, dyslipidemia, stroke, myocardial infraction and/or angina, osteoarthritis and/or rheumatoid arthritis, and diabetes mellitus

## Discussion

We aimed to examine the use of dietary supplement, the amount of nutrients consumed from foods and supplements, and their nutritional contribution to total intakes among cancer survivors and cancer-free individuals. This nationwide study found that Korean cancer survivors had a higher prevalence of dietary supplement use than cancer-free individuals. The contribution of nutrient intakes from dietary supplements to total nutrient intakes was higher among cancer survivors than cancer-free individuals. We also aimed to identify the factors associated with dietary supplement use among cancer survivors, and we found that education level above elementary school, moderate physical activity, low vegetable intake, and high circulating vitamin D levels were associated with dietary supplement use among female cancer survivors, whereas living in an urban area, no history of alcohol consumption, and low energy intake were associated with dietary supplement use among male cancer survivors.

Our study showed that 33.3% of cancer survivors reported the use of dietary supplements compared to 22.1% of cancer-free individuals. A systematic review of dietary supplement use among cancer survivors reported that 64–81% of cancer survivors used any vitamin or mineral supplements, which may be a higher proportion compared to general US adults (52%) [5]. However, previous studies that compared dietary supplement use between cancer survivors and cancer-free individuals are inconsistent [26–29]. The 2001 California Health Information Survey and the 2003 Complementary and Alternative Medicine Supplement to the California Health Information Survey found that cancer survivors were more likely to use vitamin supplements, but not minerals, herbs, and other natural products, compared to cancer-free individuals [26]. The 1987 and 1992 National Health Interview Surveys (NHIS) and Vitamins and Lifestyle (VITAL) study found no differences in the dietary supplement use rate between cancer survivors and cancer-free individuals but found that relatively high proportions of cancer survivors and cancer-free individuals used multivitamins (approximately 50% in the NHIS and over 70% in the VITAL study) [27, 28]. In the databank and biorepository (DBBR), cancer patients were less likely to use dietary supplements than cancer-free individuals [29].

Several US epidemiologic studies reported that multi-vitamins and minerals have reported as the most commonly consumed supplement type in both cancer survivors [5] and general population [30, 31]. Also, antioxidants, calcium/vitamin D, herbal/botanical, and fatty acids have reported as supplement types commonly consumed by US cancer survivors [10, 32, 33]. Consistent with these studies, we also found that multi-vitamins and minerals was the most commonly consumed supplement types in both cancer survivors (24.6% of dietary supplement users) and cancer-free individuals (33.5%). Other supplements consumed commonly were vitamin C, omega-3/fish oil, red ginseng, and calcium among cancer survivors and omega-3/fish oil, vitamin C, red ginseng, and glucosamine/chondroitin among cancer-free individuals in our study.

Limited studies have compared the amount of nutrients calculated from foods and supplements between cancer survivors and cancer-free individuals. The use of antioxidant dietary supplements in breast cancer survivors from the Women’s Healthy Eating and Living (WHEL) and general female population from the Olestra Post-Marketing Surveillance Study (OPMSS) were compared in the conference of “Free Radicals: The Pros and Cons of Antioxidants” [34]. The presenters reported that, among dietary supplement users, the median intakes of vitamin C and beta-carotene from dietary supplements were similar between the two groups, but the median supplemental vitamin E intakes were much higher among cancer survivors (268 mg/d) in the WHEL study than cancer-free individuals (34 mg/d) in the OPMSS study [34]. In our study, among overall participants, cancer survivors consumed higher amounts of calcium and vitamin C than cancer-free individuals after the addition of nutrient intakes from dietary supplements. Among dietary supplement users, however, nutrient intakes from foods only and total (foods + supplements) were similar between cancer survivors and cancer-free individuals.

We found that the percent contribution of dietary supplements to total nutrient intakes among cancer survivors who consumed dietary supplements was the highest in vitamin C (34.7%), followed by riboflavin (24.4%), and thiamin (23.9%), and these values were similar with cancer-free individuals. In French cancer survivors who consumed dietary supplements, the contribution of supplements to total vitamin C, riboflavin, and thiamine intakes were 15.6%, 9.5%, and 14.3%, respectively [3]. Although the contribution of dietary supplements was high for vitamins D (78.9%), B6 (44.4%), and E (35.4%) in that previous study [3], we could not identify the nutritional contribution of dietary supplements for those nutrients because of the limited nutrient database in our study. However, dietary supplement users tended to have higher blood vitamin D levels, suggesting the significant nutritional contribution of dietary supplements to total vitamin D intakes.

We found that the proportions of participants whose thiamin and niacin intakes below EARs were higher among cancer survivors than cancer-free individuals. The proportions of participants whose calcium intake below EARs were 61.2% for cancer survivors and 63.0% for cancer-free individuals, which was the highest rate among nutrients that we examined. The proportions of participants with nutrient intake above ULs were relatively small even after the addition of nutrient intakes from dietary supplements in both cancer survivors (< 4%) and cancer-free individuals (< 3%). A previous US study found that approximately 80% of 753 cancer survivors consumed nutrient amounts below the EARs from foods for vitamin E and magnesium, and among 559 supplement users, these values were substantially decreased after the consideration of nutrient intakes from supplements: vitamin E (81% to 12%) and magnesium (77% to 40%), but proportions of cancer survivors with consumption above ULs were less than 10% [10].

We examined the factors associated with dietary supplement use among cancer survivors. We found that the proportion of dietary supplement use varied according to the cancer site, and breast cancer had a relatively higher prevalence compared to other cancer sites. These findings were consistent with the results of a systematic review [5]. Several studies have found that, among cancer survivors, high education levels and female sex are associated with the use of dietary supplements [3, 5, 10, 32]. We found that education was also associated with the use of dietary supplements among female cancer survivors. Our study found that female cancer survivors who used dietary supplements consumed lower quantities of vegetables compared to non-users. There is evidence that a high consumption of fruits and vegetables is a predictor of the initiation and continuation of vitamin/mineral supplement use [2]. The finding that vegetable intake among supplement users was lower than in non-users warrants further research. Among male cancer survivors, dietary supplement use was associated with living in an urban area, a reduced calorie intake and, no consumption of alcohol. Korean male cancer survivors who use dietary supplements, after the diagnosis of cancer, may have healthier lifestyles compared to non-users of dietary supplements. Further studies with larger numbers of cancer survivors are necessary to identify factors associated with supplement use.

This investigation is the first study, to our knowledge, to estimate the contribution of nutrients from dietary supplements to the total nutrient intake among cancer survivors in a nationwide sample of the population in Korea. Our findings are derived from a representative sample of the community; therefore, the proportion and types of supplements used the quantities of nutrients consumed from dietary supplements, and the characteristics of dietary supplement users may be representative of Korean cancer survivors.

However, our study has several limitations. The number of cancer survivors was small. Therefore, we could not identify the characteristics of supplement users according to the cancer sites, and we could not estimate the nutritional contribution of dietary supplements among specific dietary supplement users. Detailed clinical information, including adjuvant therapy status and cancer stage, was not available. We cannot exclude the possibility that residual and unknown confounding factors may be present. There could be measurement errors from dietary and supplemental assessments, including nutrient intakes from 24-h recalls and dietary supplemental databases. Because we estimated dietary intakes from one-day 24-h dietary recalls, it may not reflect the usual diet of the participants. Furthermore, although we assumed that dietary supplement use on the previous day reflects the current usual dietary supplement use, this may not always be true. However, we found similar results in the sensitivity analysis, where we excluded participants who provided discrepant answers on their usual supplement use from the general questionnaire.

## Conclusions

Our results suggest that cancer survivors had high rate of dietary supplement use and higher contribution from dietary supplement to total nutrient intake than cancer-free individuals. Multi-vitamins and minerals were most commonly used, followed by vitamin C, omega-3/fish oil, red ginseng, and calcium among cancer survivors. We observed a higher proportion of participants whose thiamin and niacin intakes below the estimated average requirements among cancer survivors compared to cancer-free individuals. Among cancer survivors, the use of dietary supplements was associated with education levels, physical activity, vegetable intake, calorie intake, living area, and alcohol drinking status. Further studies on the use of dietary supplements among cancer survivors covering a wide range of dietary compounds are warranted. Moreover, prospective and clinical studies are necessary to clarify the association between the use of dietary supplements and cancer prognosis.

## Additional files

Additional file 1: Table S1. Dietary supplement use of study population and according to cancer sites of cancer survivors ^a^ without using sampling weight (DOCX 19 kb)

Additional file 2: Table S2. The proportions of above tolerable upper intake level (UL) among cancer survivors and cancer-free individuals (DOCX 16 kb)

## Abbreviations

25(OH) D3: 25-hydroxyvitamin D3
95% CIs: 95% confidence intervals
BMI: body mass index
DBBR: databank and biorepository
EARs: Estimated Average Requirements
FFQ: food frequency questionnaires
HDL: high-density lipoprotein
IRB: Institutional Review Board
KCDC: Korea Centers for Disease Control and Prevention
KDRIs: Dietary Reference Intakes for Koreans
KHIDI: Korea Health Industry Development Institute
KMFDS: Korean Ministry of Food and Drug Safety
KNHANES: Korea National Health and Nutrition Examination Survey
KNRLSI: Korean National Rural Living Science Institute
LS-means: Least-squares means
MEC: mobile examination center
NHIS: National Health Interview Surveys
OPMSS: Olestra Post-Marketing Surveillance Study
ORs: odds ratios
SE: standard error
ULs: Tolerable Upper Intake Levels
VITAL: Vitamins and Lifestyle
WHEL: Women’s Healthy Eating and Living
