# Supplementary material for: Dietary supplement use among cancer survivors and the general population: a nation-wide cross-sectional study
Source: BMC Cancer. 2017 Dec 28;17:891. doi: 10.1186/s12885-017-3885-1 (PMC5745960; doi:10.1186/s12885-017-3885-1)
Supplement: Supplementary file 1 — Dietary supplement use of study population and according to cancer sites of cancer survivors a without using sampling weight (DOCX 19 kb) [file 12885_2017_3885_MOESM1_ESM.docx]

Table S1. Dietary supplement use of study population and according to cancer sites of cancer survivors ^a^ without using sampling weight.

|  |  | All | | | |  | Female | | | |  | Male | | | |  |
| --- | --- | --- | --- | --- | --- | --- | --- | --- | --- | --- | --- | --- | --- | --- | --- | --- |
|  |  | n | Non-users | Users | Dietary supplement use % |  | n | Non-users | Users | Dietary supplement use % |  | n | Non-users | Users | Dietary supplement use % |  |
| Cancer-free individuals |  | 10387 | 7736 | 2651 | 25.5 |  | 6154 | 4401 | 1753 | 28.5 |  | 4233 | 3335 | 898 | 21.2 |  |
| Cancer survivors |  | 400 | 259 | 141 | 35.3 |  | 260 | 160 | 100 | 38.5 |  | 140 | 99 | 41 | 29.3 |  |
| P value ^b^ |  | <0.001 | | | |  | <0.001 | | | |  | 0.02 | | | |  |
| By site |  |  |  |  |  |  |  |  |  |  |  |  |  |  |  |  |
| Stomach |  | 71 | 49 | 22 | 31.0 |  | 27 | 16 | 11 | 40.7 |  | 44 | 33 | 11 | 25.0 |  |
| Cervix or corpus uteri |  | 67 | 39 | 28 | 41.8 |  | 67 | 39 | 28 | 41.8 |  | 0 | 0 | 0 | - |  |
| Thyroid |  | 63 | 39 | 24 | 38.1 |  | 54 | 33 | 21 | 38.9 |  | 9 | 6 | 3 | 33.3 |  |
| Breast |  | 59 | 28 | 31 | 52.5 |  | 59 | 28 | 31 | 52.5 |  | 0 | 0 | 0 | - |  |
| Colorectal |  | 52 | 40 | 12 | 23.1 |  | 27 | 22 | 5 | 18.5 |  | 25 | 18 | 7 | 28.0 |  |
| Bladder |  | 14 | 10 | 4 | 28.6 |  | 5 | 4 | 1 | 20.0 |  | 9 | 6 | 3 | 33.3 |  |
| Lung |  | 13 | 8 | 5 | 38.5 |  | 4 | 2 | 2 | 50.0 |  | 9 | 6 | 3 | 33.3 |  |
| Prostate |  | 12 | 8 | 4 | 33.3 |  | 0 | 0 | 0 |  |  | 12 | 8 | 4 | 33.3 |  |
| Liver |  | 11 | 8 | 3 | 27.3 |  | 2 | 2 | 0 | 0.0 |  | 9 | 6 | 3 | 33.3 |  |
| Renal |  | 9 | 9 | 0 | 0.0 |  | 4 | 4 | 0 | 0.0 |  | 5 | 5 | 0 | 0.0 |  |
| Larynx |  | 6 | 4 | 2 | 33.3 |  | 1 | 1 | 0 | 0.0 |  | 5 | 3 | 2 | 40.0 |  |
| Lymphoma |  | 5 | 2 | 3 | 60.0 |  | 2 | 1 | 1 | 50.0 |  | 3 | 1 | 2 | 66.7 |  |
| Skin |  | 5 | 5 | 0 | 0.0 |  | 4 | 4 | 0 | 0.0 |  | 1 | 1 | 0 | 0.0 |  |
| Ovarian |  | 4 | 3 | 1 | 25.0 |  | 4 | 3 | 1 | 25.0 |  | 0 | 0 | 0 | - |  |
| Oral cavity |  | 3 | 3 | 0 | 0.0 |  | 1 | 1 | 0 | 0.0 |  | 2 | 2 | 0 | 0.0 |  |
| Brain |  | 3 | 3 | 0 | 0.0 |  | 2 | 2 | 0 | 0.0 |  | 1 | 1 | 0 | 0.0 |  |
| Esophageal |  | 3 | 2 | 1 | 33.3 |  | 0 | 0 | 0 | - |  | 3 | 2 | 1 | 33.3 |  |
| Others^c^ |  | 17 | 13 | 4 | 23.5 |  | 6 | 4 | 2 | 33.3 |  | 11 | 9 | 2 | 18.2 |  |

^a^ Cancer survivors with multiple cancer sites were counted multiple times; 11 participants had been diagnosed with cancers at two sites and 3 participants had been diagnosed with cancers at three sites among 400 cancer survivors.

^b^ Chi-square p values were obtained using PROC FREQ to compare the proportion of dietary supplement use between cancer survivors and cancer-free individuals.

^c^ Others included 12 cancer sites (e.g. osteosarcoma, leukemia, pancreatic, and tonsillar cancer.
