# Supplementary material for: Dietary supplement use among cancer survivors and the general population: a nation-wide cross-sectional study
Source: BMC Cancer. 2017 Dec 28;17:891. doi: 10.1186/s12885-017-3885-1 (PMC5745960; doi:10.1186/s12885-017-3885-1)
Supplement: Supplementary file 2 — The proportions of above tolerable upper intake level (UL) among cancer survivors and cancer-free individuals (DOCX 16 kb) [file 12885_2017_3885_MOESM2_ESM.docx]

Table S2. The proportions of above tolerable upper intake level (UL) among cancer survivors and cancer-free individuals

| Nutrient | Above UL % (SE) | | | | |
| --- | --- | --- | --- | --- | --- |
|  | Cancer survivors | |  | Cancer-free individuals | |
|  | Foods only | Foods and supplements |  | Foods only | Foods and supplements |
| All (n) | 400 | |  | 10387 | |
| Calcium (mg/d) | 0.5 (0.5) | 0.5 (0.5) |  | 0.2 (0.05) | 0.2 (0.1) |
| Phosphate (mg/d) | 0.3 (0.3) | 0.3 (0.3) |  | 0.3 (0.1) | 0.3 (0.1) |
| Iron (mg/d) | 2.4 (0.9) | 3.3 (1.1) |  | 2.0 (0.2) | 2.7 (0.2) |
| Vitamin A (ug RE/d) | 2.1 (1.0) | 2.3 (1.0) |  | 2.3 (0.2) | 2.9 (0.2) |
| Thiamin (mg/d)^a^ | - | - |  | - | - |
| Riboflavin (mg/d)^a^ | - | - |  | - | - |
| Niacin (mg/d)^b^ | - | - |  | - | - |
| Folate (ug /d) | 0.8 (0.4) | 0.8 (0.4) |  | 1.5 (0.2) | 1.5 (0.2) |
| Vitamin C (mg/d) | 0.0 | 1.3 (0.7) |  | 0.0 | 0.6 (0.1) |
|  |  |  |  |  |  |
| Among users (n) | 141 | |  | 2651 | |
| Calcium (mg/d) | 0.0 | 0.0 |  | 0.1 (0.1) | 0.3 (0.1) |
| Phosphate (mg/d) | 0.0 | 0.0 |  | 0.3 (0.1) | 0.3 (0.1) |
| Iron (mg/d) | 0.1 (0.1) | 2.9 (1.9) |  | 2.4 (0.5) | 5.8 (0.6) |
| Vitamin A (ug RE/d) | 0.8 (0.8) | 1.4 (0.9) |  | 3.0 (0.5) | 5.6 (0.7) |
| Thiamin (mg/d)^a^ | - | - |  | - | - |
| Riboflavin (mg/d)^a^ | - | - |  | - | - |
| Niacin (mg/d)^b^ | - | - |  | - | - |
| Folate (ug /d) | 0.1 (0.1) | 0.1 (0.1) |  | 1.9 (0.4) | 1.9 (0.4) |
| Vitamin C (mg/d) | 0.0 | 3.8 (2.1) |  | 0.0 | 2.8 (0.4) |

Abbreviations: RE, retinol equivalent; DFE, dietary folate equivalents.

^a^ ULs were not determined in the Dietary Reference Intakes for Koreans 2010.

^b^ UL of niacin as nicotinic acid and nicotinamide are different. We did not calculate the proportion of above UL for niacin, because the niacin intakes as nicotinic acid and nicotinamide from dietary supplements were not classified.
